# Exploring the diversity and potential functional characteristics of microbiota associated with different compartments of *Schisandra chinensis*

**DOI:** 10.3389/fmicb.2024.1419943

**Published:** 2024-06-13

**Authors:** Wenjuan Hou, Yanping Xing, Hefei Xue, Yanchang Huang, Yutong Huang, Wenxiao Men, Yanyun Yang, Tingguo Kang, Deqiang Dou, Han Zheng, Liang Xu

**Affiliations:** ^1^School of Pharmacy, Liaoning University of Traditional Chinese Medicine, Dalian, China; ^2^State Key Laboratory of Dao-di Herbs, Beijng, China

**Keywords:** *Schisandra chinensis*, ecological community, potential functional, diversity, dominant

## Abstract

**Introduction:**

Symbiotic microbial have a significant impact on the growth and metabolism of medicinal plants. *Schisandra chinensis* is a very functionally rich medicinal herb; however, its microbial composition and diversity have been poorly studied.

**Methods:**

In the present study, the core microbiomes associated with the rhizospheric soil, roots, stems, leaves, and fruits of *S. chinensis* from six geographic locations were analyzed by a macro-genomics approach.

**Results:**

Alpha and beta diversity analyses showed that the diversity of microbial composition of *S. chinensis* fruits did not differ significantly among the geographic locations as compared to that in different plant compartments. Principal coordinate analysis showed that the microbial communities of *S. chinensis* fruits from the different ecological locations were both similar and independent. In all *S. chinensis* samples, Proteobacteria was the most dominant bacterial phylum, and Ascomycota and Basidiomycota were the most dominant fungal phyla. *Nitrospira, Bradyrhizobium, Sphingomonas*, and *Pseudomonas* were the marker bacterial populations in rhizospheric soils, roots, stems and leaves, and fruits, respectively, and *Penicillium, Golubevia*, and *Cladosporium* were the marker fungal populations in the rhizospheric soil and roots, stems and leaves, and fruits, respectively. Functional analyses showed a high abundance of the microbiota mainly in biosynthesis.

**Discussion:**

The present study determined the fungal structure of the symbiotic microbiome of *S. chinensis*, which is crucial for improving the yield and quality of *S. chinensis*.

## 1 Introduction

*Schisandra chinensis* is the dried fruit of *S. chinensis*, belonging to the family Magnoliaceae, and is known as “Bei wuwei” (Chinese Pharmacopoeia Commission, 2020). *S. chinensis* has been used as a traditional herbal medicine in Asian countries such as China, Korea, Japan, and Russia. It exhibits diverse pharmacological activities, including antioxidative, antitumor, antiobesity, anti-inflammatory, cardioprotective, and hepatoprotective effects. It is used to treat autoimmune diseases, cardiovascular diseases, acne, and neurological disorders (Nagappan et al., 2018). Schisandrin B and schisandrol are the representative lignans of *S. chinensis*. Schisandrin B is the most important constituent of *S. chinensis*, and there is increasing evidence that this compound possesses various pharmacological properties such as anticancer, anti-inflammatory, hepatoprotective, and antimicrobial activities. Several studies have demonstrated the anti-inflammatory effects of these lignans (Zhang et al., 2021). *S. chinensis*, as a functional food in China, is rich in unsaturated fatty acids, minerals, vitamins, and proteins, and it is used as a tonic and sedative in Chinese medicine. Schisandrin A is one of the lignans isolated from the dried fruits of *S. chinensis* (Wang X. et al., 2022; Wang Q. et al., 2022). It has a wide range of pharmacological effects, including antioxidation, apoptosis inhibition, and immunomodulatory effects (Zong et al., 2021). Because of the high market demand for *S. chinensis*, wild-type *S. chinensis* alone cannot meet the requirement; consequently, more planting areas of *S. chinensis* have been developed.

The differences in plant growth and chemical composition are closely associated with its geographic location. The microbial community in the plant’s environment also affects the growth of plants. The inner circle of plants is colonized by endophytes, which are complex microbial communities and microbial species that colonize the inner surfaces of plants for some part of their life cycle. Endophytic fungi living in plant tissues are found in almost all plant species (Saikkonen et al., 1998). During the evolutionary process, endophytic microbial communities have formed mutualistic relationships with plants: they receive nutrients and protection from host plants, which in turn may benefit from enhancing competitive ability and resistance to herbivores, pathogens, and various abiotic stresses (Saikkonen et al., 1998; de Melo Pereira et al., 2012).

The diversity and composition of rhizospheric microbial communities are critical for maintaining soil quality and plant health (Berendsen et al., 2012). Plants provide a nutrient-rich environment for the growth and development of endophytic bacteria and fungi (Li et al., 2021). Endophytes can significantly affect the quality and quantity of medicinal plant raw materials by regulating plant immunity and influencing plant metabolism (Wang et al., 2021). Furthermore, microbial metabolites, which are key signaling mediators for microorganisms, plants, and microbial-plant interactions, are important for revealing their functional mechanisms (Li et al., 2022).

Plant-associated microorganisms, particularly endophytes, have recently received considerable attention because of growing awareness of the critical role of host-associated microbiota in the optimal functioning and performance of their host. Although some functions of endophytes have been determined primarily through laboratory assays, genome prediction, and macro-genomic analyses, our understanding of plant activities remains limited, given the diversity of microenvironments and the dynamics of environmental conditions (Compant et al., 2021).

In the present study, we investigated the diversity and composition of the microbiome associated with *S. chinensis* through the internal transcribed spacer (ITS) region and 16S ribosomal RNA (16S rRNA) sequence analysis. The study aimed to identify the microbiome of *S. chinensis* and to develop a method for identifying microbial species associated with the quality and growth of *S. chinensis*.

## 2 Materials and methods

### 2.1 Sample research topic and processing

In September 2023, samples of rhizospheric soil, roots, stems, leaves, and fruits of *S. chinensis* (*n* = 180) were collected from six geographic locations (Qianshan, Zhuanghe, Xinbin, Fengcheng, Kuandian, and Huanren) in Liaoning Province, China (Supplementary Table S1). *S. chinensis* samples from Qianshan were wild-type *S. chinensis* (*n* = 30), and the remaining samples from the other locations were field-planted *S. chinensis* (*n* = 150). Except wild samples were uncertain, the cultivated samples of *S. chinensis* we have collected are in the range of 4–5 years old (Supplementary Table S1). Three individual *S. chinensis* plants were randomly selected from each plot (3–5 m distant from each other). Healthy, intact leaves (10–20 g) were selected from the plants and placed in a clean and dry zip-lock bag. Subsequently, fruits (50–100 g) and stems (20–30 g) were collected from the same plant. Finally, the rhizospheric soil (defined as soil tightly attached to the plant roots) and roots (20–30 g) of the same plant were collected. All samples were temporarily stored in clean and dry zip-lock bags. These bags were placed in an ice pack and promptly delivered to the laboratory for analysis. First, fresh plant samples were rinsed repeatedly with tap water to completely remove soil debris and surface microorganisms, until the liquid rinsed off was clear. Roots and stems were cut into 2 cm sections and surface sterilized by soaking in 75% alcohol for 3 min. The leaves and fruits were soaked in alcohol for 2 min, washed three times with sterile distilled water, and drained on a sterile filter paper. They were placed in prepared sterile bags. The rhizospheric soil was not sterilized; it was collected (1.5 g) using sterilized clean gloves and placed in sterile bags. These bags were kept at −80°C until analysis (Qin et al., 2022).

### 2.2 DNA extraction and PCR amplification and sequencing

For analysis, the samples were removed from the refrigerator, and an appropriate amount of sample (0.2–0.5 g) was immediately added to a centrifuge tube containing the extraction lysate for grinding and processing. Following the completion of pretreatment, a kit was used to extract nucleic acids from the samples. The extracted DNA was subjected to 0.8% agarose gel electrophoresis for molecular size determination and quantified with a Nanodrop NC2000 spectrophotometer (Thermo Fisher Scientific, Waltham, MA, United States). Based on the sequenced regions (V5–V7 for symbiotic bacteria; ITS1 for symbiotic fungi), specific primers with barcode were used (16S rRNA V5–V7: F: AACMGGATTAGATACCCKG, R: ACGTCATCCCCACCTTCC; ITS1(b): F: CTTGGTCATTTAGAGGAAGTAA, R: GCTGCGTTCTTCATCGATGC) for amplification and sequencing (Horton et al., 2014; Xing et al., 2023). The PCR reaction mixture included the following components: 0.25 μL of Q5 high-fidelity DNA polymerase, 5 μL of 5× reaction buffer, 5 μL of 5× high GC buffer, 2 μL of dNTP (10 mM), 2 μL of template DNA, 1 μL each of forward and reverse primers (10 μM), and 8.75 μL of ddH_2_O. The thermal cycling program was as follows: (1) initial denaturation at 98°C for 5 min, (2) 30 cycles of denaturation at 98°C for 30 s, annealing at 55°C for 45 s, and extension at 72°C for 45 s, and (3) final extension at 72°C for 5 min, followed by storage at 12°C. The PCR products were purified using VAHTSTM DNA Clean Beads (Vazyme, Nanjing, China) and quantified with Quant-iT PicoGreen dsDNA assay kit (Invitrogen, Carlsbad, CA, United States). After the individual quantification steps, amplicon aliquots were pooled and sequenced in pairs of 2 × 250 bp by using the NovaSeq 6000 SP reagent kit (500 cycles; Shanghai Personal Biotech, Shanghai, China). The library was constructed using the TruSeq Nano DNA LT library prep kit (Illumina). Next, we performed 2 × 250 bp bipartite sequencing for confirmed libraries on an Illumina NovaSeq machine by using the NovaSeq 6000 SP reagent kit (500 cycles).

### 2.3 Sequencing analysis

The biological information of the microbiome was analyzed using QIIME2 version 2019.4 (Bolyen et al., 2018). Raw sequence data were decoded using the demux plugin, and primer excision was performed using the cutadapt plugin (Martin M. 2011). By using the DADA2 plugin, further quality filtering, denoising, splicing, and chimerism removal processes were performed on the sequences to generate feature amplicon sequence variants (ASVs) and abundance data tables (Callahan et al., 2016).

Taxonomic information corresponding to each ASV was obtained using the MaarjAM database (Koljalg et al., 2013), and the ASV feature sequences were compared with the reference sequences in the database (Bokulich et al., 2018). Based on the results of ASV delineation and taxonomic status identification, the species-specific composition of each sample at each taxonomic level was obtained. Taxonomic composition analyses were performed for different taxonomic levels, including phylum, order, family, genus, and species. For alpha-diversity analysis, the following two diversity indices, namely Shannon index (Shannon, 1948a,b; Shannon et al., 2003) and Simpson index (Simpson, 1949), were calculated for each sample by using QIIME2 software, and box plots were drawn to compare the abundance and homogeneity of ASVs among the samples. Beta-diversity analyses were performed to determine changes in microbial community structure between samples (Lozupone and Knight, 2005; Lozupone et al., 2007), and the results were analyzed by principal coordinate analysis (PCoA) with the Bray-Curtis method (Bray and Curtis, 1957) and hierarchical clustering (UPGMA) (based on the Bray-Curtis method, unweighted UniFrac distance) (Jaccard, 1908; Lozupone and Knight, 2005) and weighted UniFrac distance method (Lozupone et al., 2007) for visualization. For determining species relationships, Cisco map analysis was performed using Genescloud tools, a free online platform for data analysis.^1^ QIIME2 software was used to obtain composition and abundance tables for each sample at the six taxonomic levels of phylum, class, order, family, genus, and species, and the results of the analyses were presented as random forests. The linear discriminant analysis effect size (LEfSe) method was used to detect categorical units with considerable differences between the groups. The association network was constructed using the SparCC analysis method; the pseudocount value in SparCC was set as 10^−6^. Metabolic functions of the microflora were predicted by PICRUSt2 (systematic genetic investigation of communities by reconstruction of unobserved states) on the MetaCyc database^2^ for prediction. Comparative analyses of metabolic pathways in different samples were also performed using STAMP software (Gavin et al., 2019).

## 3 Results

### 3.1 Illumina sequencing analysis

A total of 17,929,590 raw reads were obtained from 180 samples. After removing low-quality sequences, 9,320,707 high-quality sequence reads for bacteria were obtained from 90 samples. A total of 4,771,995 ASVs were assigned, ranging from 78,420 to 134,393 per sample (Supplementary Table S2). Furthermore, after removing low-quality sequences, 7,505,123 high-quality sequence reads for fungi were obtained from 90 samples. A total of 6,702,814 ASVs were assigned, ranging from 70,988 to 116,313 per sample. The slopes of the sparsity curves were flat at different similarity thresholds, thus indicating that the sequencing results were sufficiently diverse to reflect the diversity of the samples (Supplementary Figures S1A,C). This observation was also confirmed by the species accumulation curves (Supplementary Figures S1B,D).

### 3.2 Species diversity analysis of symbiotic microorganisms

**Figure 1:**
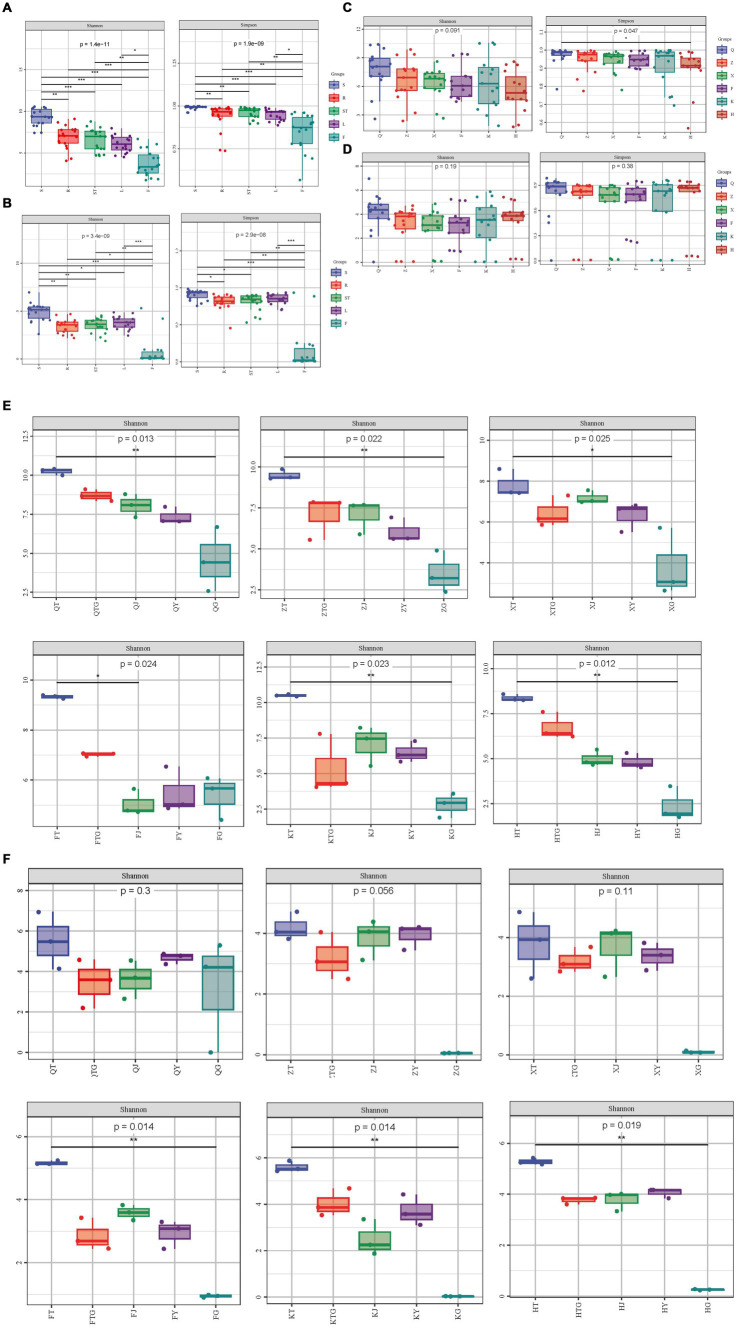
Microbial diversity analysis. **(A)** Shannon and Simpson diversity indices of bacteria in the different organs of *S. chinensis*. **(B)** Shannon and Simpson diversity indices of fungi in the different organs of *S. chinensis*. **(C)** Shannon and Simpson diversity indices of bacteria in *S. chinensis* from different geographic locations. **(D)** Shannon and Simpson diversity indices of fungi in *S. chinensis* from different geographic locations. **(E)** Shannon index of bacteria in the different organs of *S. chinensis* from the six geographic locations. **(F)** Shannon index of fungi in the different organs of *S. chinensis* from the six geographic locations.

Higher values of the Shannon index/Simpson index in the alpha-diversity analysis indicate higher diversity of the community. The results of the significance analysis of diversity indices of bacteria showed that the Shannon and Simpson indices of rhizospheric soil bacteria of *S. chinensis* were significantly higher than those of bacteria of roots, stems, leaves, and fruits (Figures 1A,E). This shows that the soil of *S. chinensis* is richer in bacteria and has a higher species uniformity. Here, we noted the selective enrichment of soil bacteria by *S. chinensis* plants. Highly significant differences were observed in the Shannon index and Simpson index of each site in different ecological niches; this finding indicated differences in the diversity and evenness of symbiotic bacteria in *S. chinensis* (Figures 1A,E). Furthermore, the diversity indices of different organs bacteria showed a decreasing trend in the order of roots > stems > leaves > fruits, thus indicating that the diversity of bacteria in each tissue part of *S. chinensis* gradually decreased from underground to aboveground; additionally, symbiotic bacteria showed more regular distribution in different ecological sites (Figure 1E). The Shannon index of wild *S. chinensis* in Qianshan was the highest among those of the six areas. The diversity index values of *S. chinensis* from Qianshan, Zhuanghe, and Huanren showed a decrease in the order of roots > stems > leaves > fruits (Figure 1C). The results of significance analysis of diversity indices of fungi showed that the values of Shannon and Simpson indices of fungi in the rhizospheric soil of *S. chinensis* were significantly higher than those of fungi of roots, stems, leaves, and fruits (Figures 1B,F). The soil of *S. chinensis* had a rich and more homogeneous content of fungal species. The highly significant differences in the Shannon index and Simpson index among the different ecological niches of each site indicated differences in the diversity and evenness of fungi of *S. chinensis* (Figures 1B,F). In addition, when the data from six geographical locations were combined, there was no significance between the provenances of *S. chinensis*. Of the six origins, only Fengcheng, Kuandian, and Huanren *S. chinensis* had highly significant differences between the rhizospheric soils and the different organs fungal communities (Figure 1D). A comparison of wild-type and cultivated *S. chinensis* revealed that the Shannon index and Simpson index of wild-type *S. chinensis* from Qianshan were higher than those of cultivated *S. chinensis*, thus indicating that the uniformity and diversity of the fundal community of wild-type *S. chinensis* were higher than those of cultivated *S. chinensis*. Furthermore, the diversity of the fungal community of fruits and leaves of wild-type *S. chinensis* from Qianshan was higher than that of fruits and leaves of other cultivated *S. chinensis* (Figure 1D).

**Figure 2:**
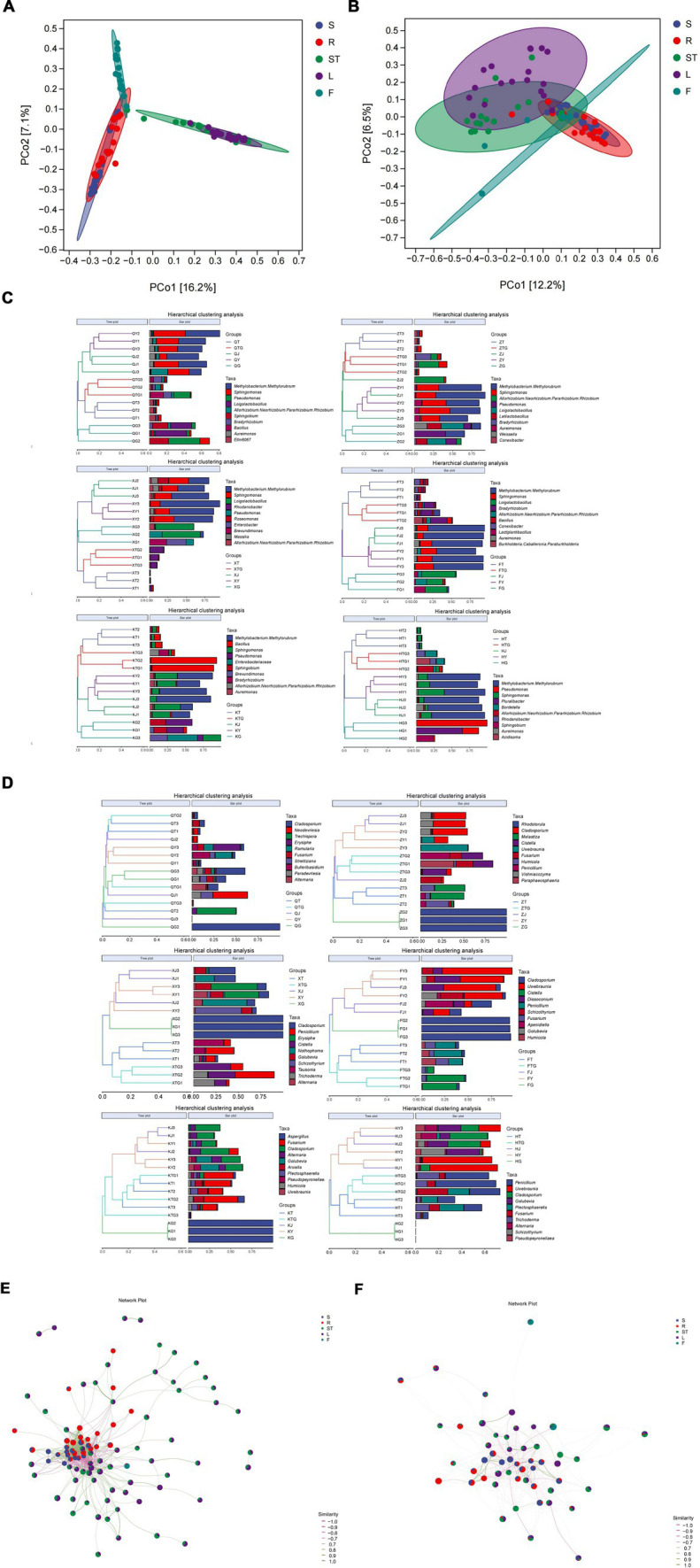
Beta-diversity analysis of the microbial community and community composition of *S. chinensis*. **(A,B)** Show the results of principal coordinate analysis of Bray-Curtis distance matrices for bacterial and fungal communities in different ecological niches. **(C,D)** Show systematic clustering of the relative abundance of top 10 bacterial and fungal communities from different ecological niches at the genus level. **(E,F)** show bacterial **(E)** and fungal **(F)** association network diagrams of the different organs of *S. chinensis*.

The rhizospheric soil bacterial community of *S. chinensis* was clustered with the bacterial community of the roots, the bacterial communities of the stem and leaf parts were clustered together, and the bacterial community of fruits formed a separate cluster (Figure 2A). A similar finding was observed for the fungal communities of *S. chinensis*. The rhizospheric soil fungi of *S. chinensis* were clustered together with the fungal community of roots; a few fungal communities of the stems and leaves were clustered together, while most fungal communities of the stems and leaves were clustered separately; and most fungal communities of the fruits were clustered separately, with only a few fungal communities intertwined with those of the other organs (Figure 2B). This result was confirmed by the correlation network diagram between the different organs of *S. chinensis* (Figures 2E,F). This finding indicates a correlation between the different organs of *S. chinensis*, but not all of the symbiotic microbial community follow the order from underground to above ground. To investigate the differences in the abundance of symbiotic microbial communities observed in the samples, a clustering tree of the samples was constructed by clustering analysis of the different samples. Hierarchical cluster analysis was performed using the Bray-Curtis distance method. The clustering results were integrated and presented with the relative abundance of species at the genus level for each sample. Consistent with the results of the PCoA analysis for the clustering of symbiotic communities in the rhizospheric soil, it was observed that the bacterial communities in *S. chinensis* fruits differed greatly from those in the other parts of the plant (Figure 2C). The results of the hierarchical clustering analysis of fungi were similar to the results of PCoA, with an overall view of most roots clustered together in the rhizospheric, and the fungal communities of other organs intertwined (Figure 2D). Hierarchical clustering analysis was performed with the weighted UniFrac distance matrix and the unweighted UniFrac distance matrix. The results showed that the clustering tree of *S. chinensis* was divided into three major branches: rhizospheric soil bacteria and root bacteria, stem bacteria and leaf bacteria, and fruit bacteria (Supplementary Figures S3A,B). These results were further validated.

A comparison of the same organ from different origins showed that the bacterial community of the leaves of wild-type *S. chinensis* from Qianshan was separate from that of cultivated *S. chinensis* and formed a single branch. The fungal communities of the roots and stems of wild-type *S. chinensis* from QianShan were separate from those of cultivated *S. chinensis* and formed a separate branch (Supplementary Figures S4A,B). The results of rhizospheric soil analyses of *S. chinensis* from different origins showed that the rhizospheric soil and root bacterial communities of wild-type *S. chinensis* from Qianshan and cultivated *S. chinensis* from Kuandian had a high degree of similarity. For the fungal community (Supplementary Figures S4C,D), the fruit samples of *S. chinensis* from Huanren, Zhuanghe, and Kuandian were clustered on one branch each. Overall, bacterial community composition was determined by different ecological niches, with bacterial communities from the same geographical location in the same ecological niche being more similar in composition. Fungal communities did not show this phenomenon, and its composition was not strictly influenced by ecological niche and geographical location (Supplementary Figures S4C,D).

### 3.3 Analysis of ASVs in the rhizospheric soil and different organs of *Schisandra chinensis*

**Figure 3:**
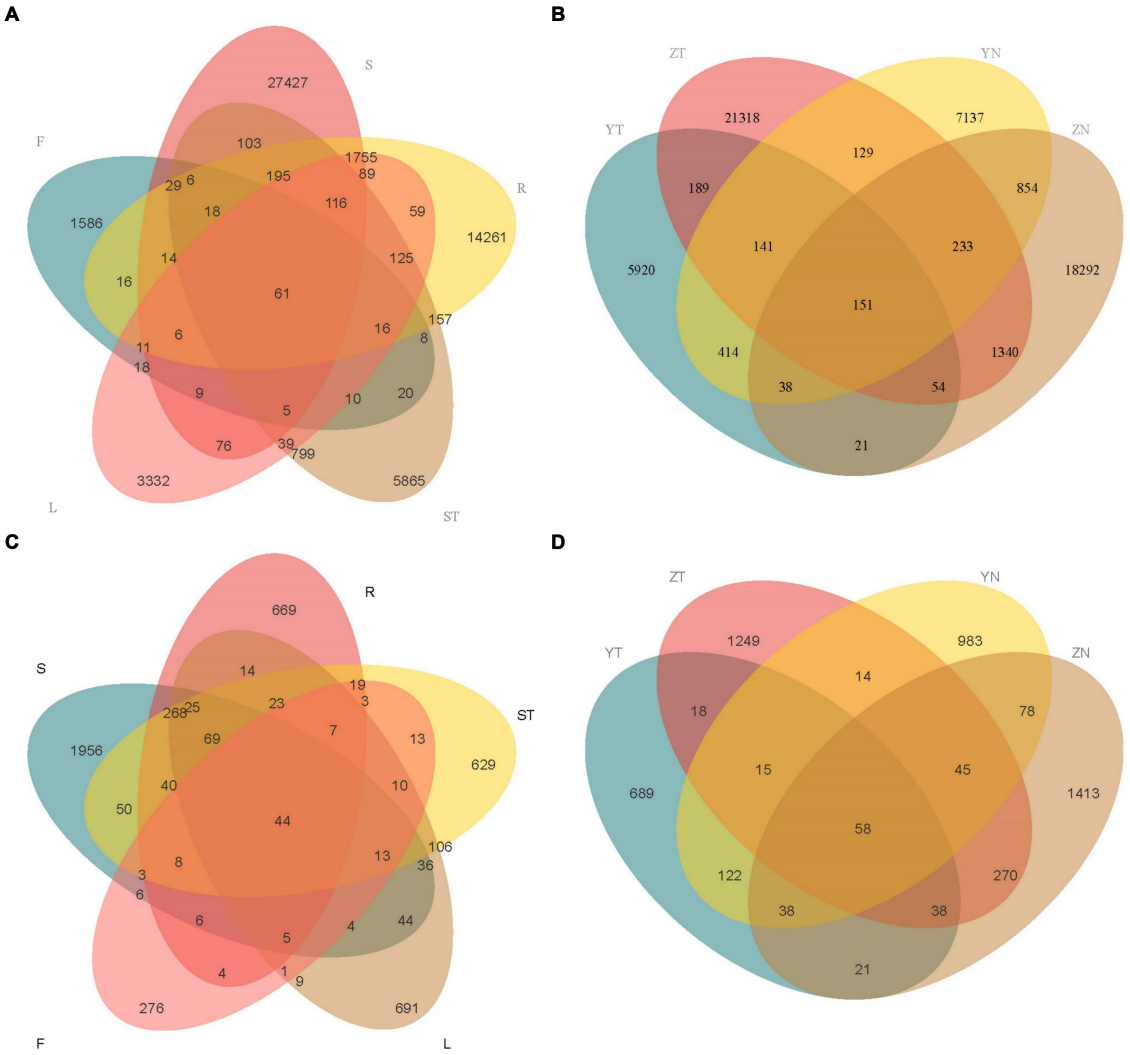
Venn diagram analysis of the number of overlapping ASVs across the different compartments of *S. chinensis*. **(A,B)** Number of overlapping ASVs in bacteria and fungi in the rhizospheric soil, roots, stems, leaves, and fruits of *S. chinensis*. **(C,D)** Number of overlapping ASVs in rhizospheric soil and symbiotic bacteria and fungi of wild-type and cultivated *S. chinensis*. Rhizospheric soil: S; Root: R; Stem: ST; Leaf: L; Fruit: F; Wild-type *S. chinensis* rhizospheric soil: Y; Wild-type *S. chinensis* bacteria/fungi in other organs: YN; Cultivated *S. chinensis* rhizospheric soil: Y; Cultivated *S. chinensis* bacteria/fungi in other organs: YN.

Based on the ASV results obtained from clustering analysis and the study requirements, the shared and unique ASVs among the different samples were analyzed. Roots and rhizospheric soil had the most specifically enriched ASVs among the bacterial communities (14,261 and 27,427, respectively) (Figure 3A). Furthermore, rhizospheric soil and leaves had the most specifically enriched ASVs among the fungal communities (1,956 and 691, respectively) (Figure 3B). The symbiotic bacteria of *S. chinensis* contained 61 core ASVs, with the roots (R), stems (ST), leaves (L), and fruits (F) containing 77 ASVs from the symbiotic bacteria (Figure 3A). The symbiotic fungi of *S. chinensis* contained 44 core ASVs, with the roots (R), stems (ST), leaves (L), and fruits (F) containing 51 ASVs from the symbiotic fungi (Figure 3B).

The rhizospheric soil bacteria of wild-type *S. chinensis* and cultivated *S. chinensis* had 5,920 and 21,318 specifically enriched ASVs, respectively, with 535 ASVs common between them. The bacteria in other organs of wild-type *S. chinensis* and cultivated *S. chinensis* had 7,137 and 18,292 specifically enriched ASVs, respectively, with 1,276 ASVs common between them (Figure 3C). The rhizospheric soil fungi of wild-type and cultivated *S. chinensis* had 689 and 1,249 specifically enriched ASVs, respectively, with a total of 129 ASVs common between them. The fungi in other organs of wild-type *S. chinensis* and cultivated *S. chinensis* had 983 and 1,413 specifically enriched ASVs, respectively, with 219 ASVs common between them (Figure 3D).

**Figure 4:**
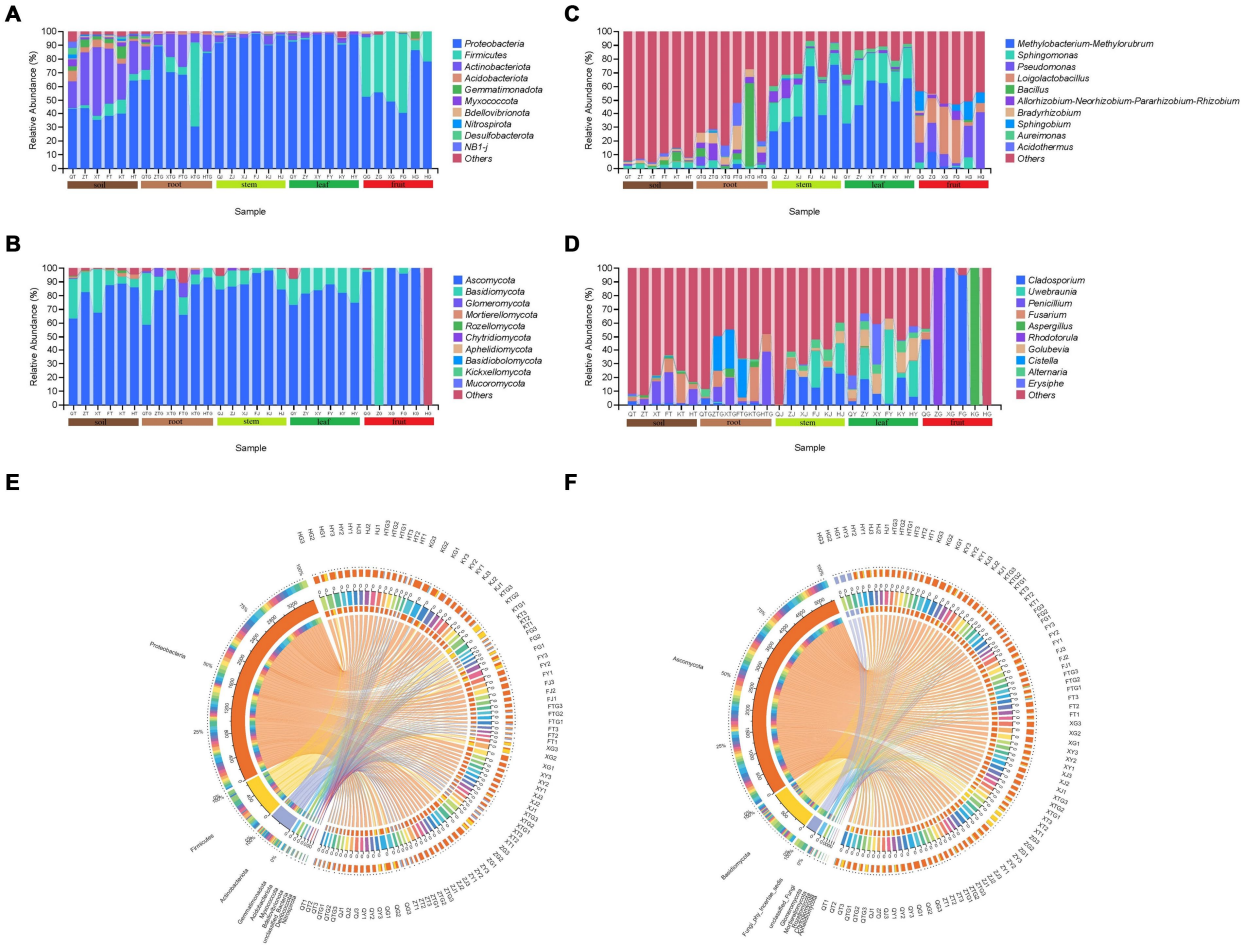
Histogram of the relative abundance of symbiotic and rhizospheric soil microbial communities of *S. chinensis* at the phylum (top 10) and genus (top 10) level. **(A,B)** Represent bacterial and fungal communities at the phylum level; **(C,D)** represent bacterial and fungal communities at the genus level. **(E,F)** Cisco map of species relationships. **(E,F)** Represent the bacterial and fungal communities, respectively, of *S. chinensis*.

The results of ASV annotation and relative abundance showed that in the bacterial community, Proteobacteria was the most dominant phylum in all samples (Figures 4A,E). The abundance of Proteobacteria (44.56%) was slightly lower and that of Actinobacteriota (33.06%) was slightly higher in the rhizospheric soil of *S. chinensis* as compared to the abundance of the bacteria in other organs. The abundance of Proteobacteria was higher in roots (68.21%), stems (94.64%), and leaves (95.15%) than in fruits (60.58%), and that of Firmicutes (37.05%) was higher in fruits than in roots (14.35%), stems (0.52%), and leaves (0.66%). Acidobacteriota, Myxococcota, Bdellovibrionota, Nitrospirota, Desulfobacterota, and NB1-j exhibited very low relative abundance content in fruits. The relative abundance of Firmicutes showed a decrease in the following order: rhizospheric soil (33.06%), roots (13.89%), stems (2.95%), leaves (1.85%), and fruits (0.50%) (Figure 4A). Among the fungal communities, Ascomycota was the most dominant phylum among all the samples (Figures 4B,F). *Ascomycota* (79.32%) and Basidiomycota (16.31%) showed slightly higher abundance in the rhizospheric soil of *S. chinensis* than the fungi in other organs. The abundance of Ascomycota was higher in roots (80.35%), stems (89.71%), and leaves (80.74%) than in fruits (65.56%). The relative abundance of Glomeromycota (3.49%) and Mortierellomycota (0.23%) was higher in roots than in other organs, and stems had a high relative abundance of Chytridiomycota (0.26%). The relative abundance of Ascomycota showed a decrease in the following order: rhizospheric soil (16.31%), roots (13.47%), and stems (8.71%); however, *Ascomycota* was enriched in leaves (17.84%) and fruits (17.57%) (Figure 4C).

At the genus level, the dominant genus (top 10) in *S. chinensis* was *Methylobacterium-Methylorubrum* with the highest relative abundance, followed by *Sphingomonas*. Among the bacteria in different organs, *Pseudomonas* (18.45%) and *Loigolactobacillus* (18.98%) showed higher abundance content in fruits than in other ecological niches. In contrast, *Sphingomonas* showed higher abundance (24.57%) in leaves than in other ecological niches. Stems (48.31%) and leaves (53.51%) exhibited a high abundance of *Methylobacterium-Methylorubrum*. The abundance of *Bacillus* in roots was 12.30%. The top 10 rhizospheric soil bacteria had a lower abundance than the bacteria in other organs (Figure 4C). In the fungal community, the dominant genus (top 10) in *S. chinensis* was *Cladosporium* with the highest relative abundance, followed by *Uwebraunia*. Among the fungi in different organs, *Cladosporium* (40.49%), *Aspergillus* (16.68%), and *Rhodotorula* (16.64%) had higher abundance content in fruits; *Uwebraunia* (17.93%), *Golubevia* (11.78%), and *Erysiphe* (8.49%) showed higher abundance content in leaves; *Alternaria* (4.69%) showed higher abundance in stems; and *Fusarium* (8.96%), *Penicillium* (12.53%), and *Cistella* (13.53%) exhibited higher abundance in roots. The fungal abundance content of the top 10 rhizospheric soil fungi was lower than that of fungi in other organs (Figure 4D).

A comparison between wild-type and cultivated *S. chinensis* revealed that the top 10 microbial taxa composition in both varieties was similar, with differences only in relative abundance. For example, among the symbiotic bacteria, the relative abundance of *Methylobacterium-Methylorubrum* (15.34%) was lower in wild-type *S. chinensis* than in cultivated *S. chinensis* (28.50%) (Figure 4C; Supplementary Figure S2A). Similarly, among the symbiotic fungi, the relative abundance of *Fusarium* was lower in wild-type *S. chinensis* (14.00%) than in cultivated *S. chinensis* (18.24%) (Figure 4D; Supplementary Figure S2C). The microflora of *S. chinensis* from different geographical locations showed considerable differences in relative abundance. For example, *Methylobacterium-Methylorubrum* (28.54%) exhibited a higher relative abundance in *S. chinensis* from Fengcheng (Figure 4C; Supplementary Figure S2B), while *Fusarium* (26.03%) showed a higher relative abundance in *S. chinensis* from Xinbin (Figure 4D; Supplementary Figure S2D).

### 3.4 Analysis of differences in different organs and rhizospheric soil microbial species and marker species

**Figure 5:**
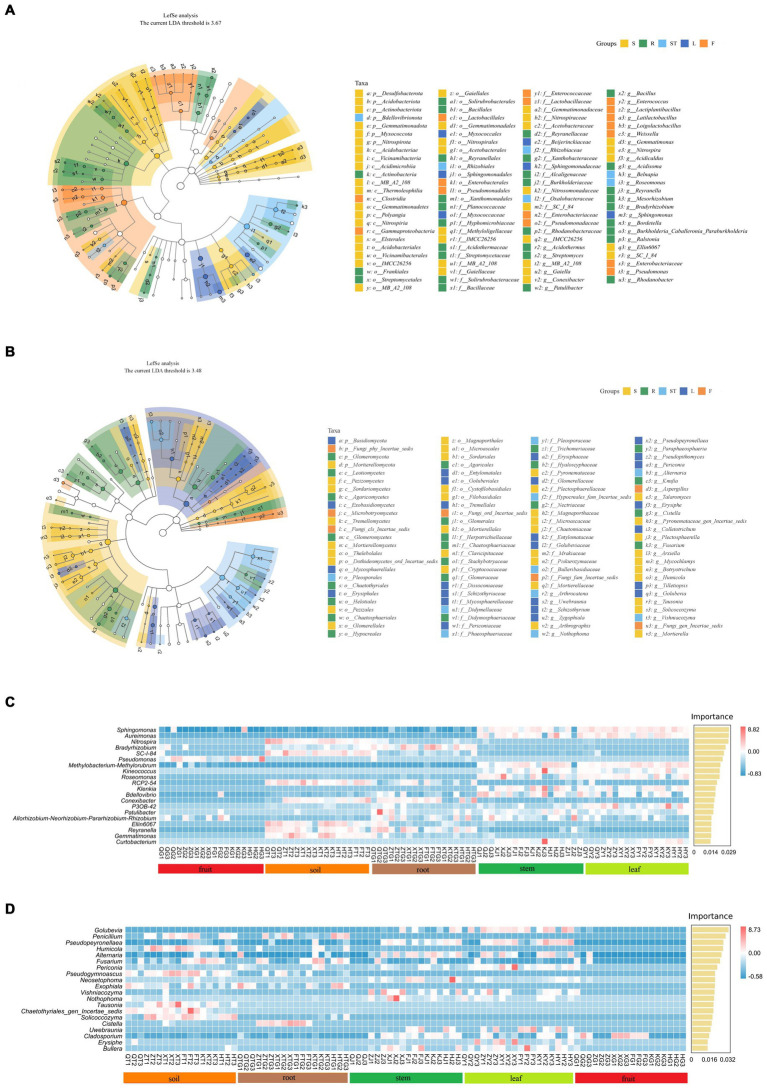
Biomarker analysis of rhizospheric soil, roots, stems, leaves, and fruits of *S. chinensis*. **(A,B)** Show the evolutionary branching diagram of *S. chinensis* based on the LEfSe assay. Bacterial community **(A)** and fungal community **(B)**. **(C,D)** Random forest analysis was used to represent the biomarkers of *S. chinensis*. Bacterial community **(C)** and fungal community **(D)**.

To analyze the effects of different plant organs on the composition of microbial communities, we performed LEfSe analysis to determine the relationship between microbial communities and plant organs. LDA effect size analyses showed higher significant differences that biomarkers in the symbiotic microbial community. The bacterial community of *S. chinensis* showed a significant increase in the proportions of Actinobacteriota, Thermooleophilic, *Acidimicrobiia*, *Acidobacteriota*, *Myxococcota*, *Acetobacteriales*, and *Acetobacteraceae* in the rhizospheric soil. The proportions of Gammaproteobacteria, Lactobacillales, Enterobacterales, Enterobacteriaceae, and Lactobacillaceae were significantly increased in fruits; Actinobacteria, Xanthomondales, Solirubrobacterales, Bacillales, Bacillaceae, and Xanthobacteracea were enriched in roots; Rhizobiales and Rhizobiaceae were increased in stems; and Sphingomonadales, Sphingomonadaceae, Beijerinckiaceae, and Sphingomonas were significantly increased in leaves. Overall, Actinobacteriota, Gammaproteobacteria, and Rhizobiales were significantly enriched in the inner plant circle (Figure 5A). Among the fungal community of *S. chinensis*, the proportions of *Sordariomycetes, Sordariales, Chateomiaceae, and Plectosphaerellaceae were significantly increased in the rhizospheric soil, Microbotryomycetes and Aspergillus showed a significant increase in fruits; Basidiomycota, Tremellales, Mycosphaerellaceae, Dissoconiacea, Mycosphaerellales, Uwebraunia, and Colletotrichum were significantly increased in leaves; Pleosporales, Didymellaceae, Phaeosphaeriaceae, Bulleribasidiaceae, Hypocreales-fam-Inceretae-sedis, and Vishniacozyma were significantly enriched in stems; and Leotiomycetes,* Agaricomycetes, Hypocreales, Helotiales, Chaetothyriales, Nectriaceae, Agaricales, and Fusarium were significantly increased in the roots. Overall, the inner plant circle showed significant enrichment of Basidiomycota, Pleosporales, Hypocreales, and Sordariomycetes (Figure 5B).

To better characterize the influence of and geographic location on microbial community composition at the species level, dominant and biomarker taxa were identified for each compartment area by random forest analysis. The differences were considerably significant at the genus level mainly in the rhizospheric soil and inner circle communities. In the bacterial community, eight major marker populations were identified in the rhizospheric soil, among which *Nitrospira* was the significant marker population. *Pseudomonas* was the marker population in fruits, and *Bradyrhizobium* was the major marker population in several root samples. Twelve major marker populations were identified in stem and leaf samples, among which *Sphingomonas* was the major marker population (Figure 5C). In the fungal communities, eight major marker populations were identified in the rhizospheric soil, with *Penicillium* as the significant marker population. *Penicillium* was also the major marker population in the majority of root samples. Eleven major marker populations were identified in stem and leaf samples, with *Golubevia* as the major marker population in some of the stem samples and the significant marker population in leaves. There were few major marker populations in fruits, and *Cladosporium* was a significant marker population in some of the fruit samples (Figure 5D).

### 3.5 Functional metagenomic analysis

**Figure 6:**
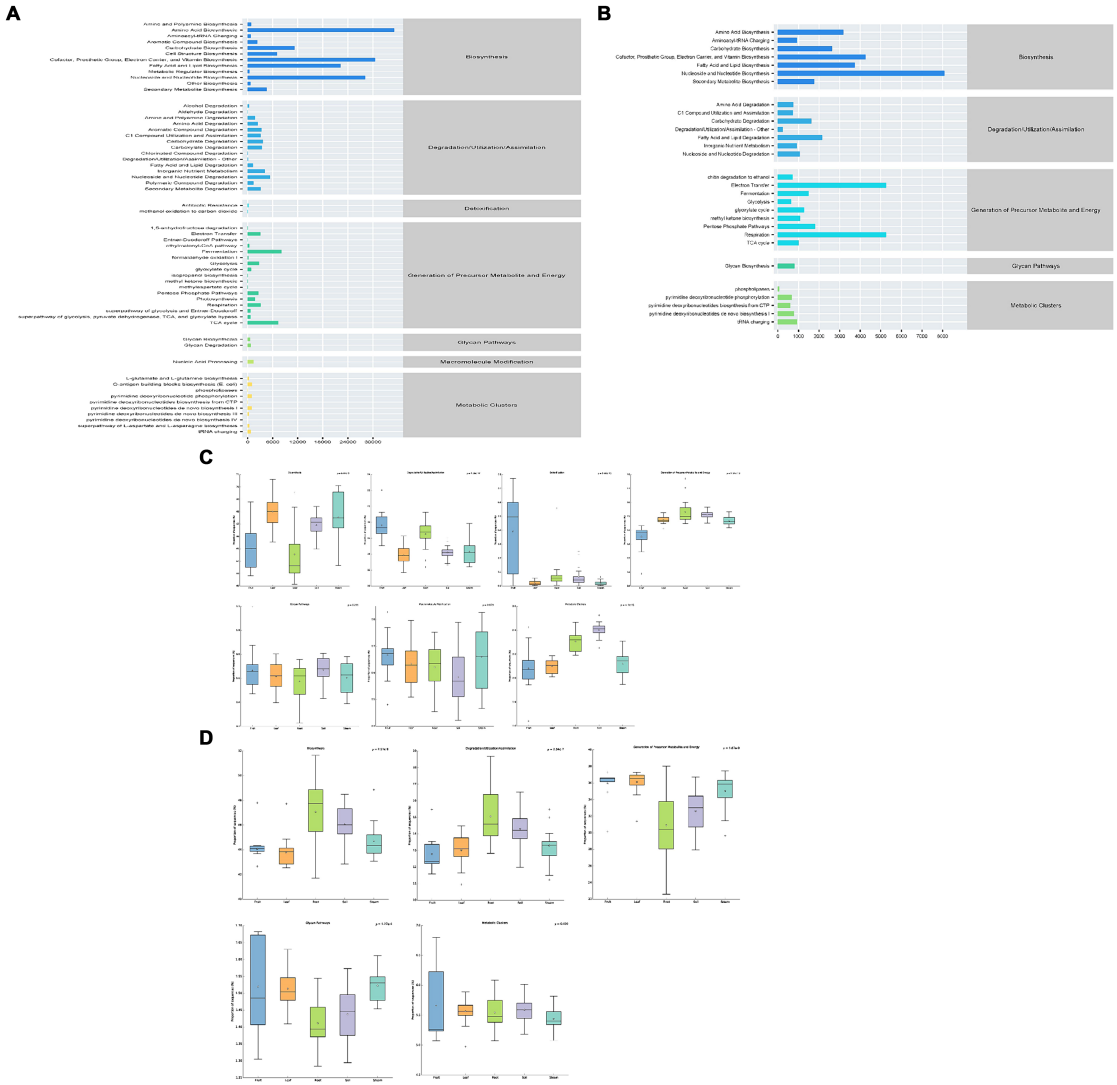
Statistical map of metabolic pathways for the predictive analysis of rhizospheric soil microbial and endosymbiotic microbial function in *S. chinensis.* **(A,C)** Represent bacterial communities. **(B,D)** Represent fungal communities.

The microbiomes of symbiotic bacteria of *S. chinensis* were analyzed on the basis of 16S rRNA amplicon sequencing results. The functional profiles of the bacterial core microbiome were predicted using PICRUST. A total of 59 pathways were identified. The main functions of specific rhizospheric and endosymbiotic bacteria with high abundance were biosynthesis, degradation/utilization/assimilation, detoxification, generation of precursor metabolites and energy, glycan pathway, macromolecule modification, and metabolic clusters (Figure 6A). Biosynthesis and production of precursor metabolites and energy were the predominant functions of the bacterial community in leaves; degradation/utilization/assimilation, detoxification, and macromolecule modification were the predominant functions of the bacterial community in fruits; the glycan pathway was the predominant metabolic function of the bacterial community in stems; and the rhizospheric soil bacterial community of *S. chinensis* showed metabolic clustering as the main function (Figure 6C).

Based on the sequencing results of ITS gene amplicons, the microbiomes of symbiotic fungi of *S. chinensis* were analyzed, and the functional profiles of the core fungal microbiome were predicted using PICRUST. A total of 29 pathways were identified. The main functions of specific rhizospheric and symbiotic fungi with high abundance were biosynthesis, degradation/utilization/assimilation, generation of precursor metabolites and energy, glycan pathway, and metabolic clustering (Figure 6B). Fungal communities in *S. chinensis* roots showed biosynthesis and degradation/utilization/assimilation as the main function, those in fruits showed generation of precursor metabolites and energy as the main functions, those in stems showed glycan pathway and metabolic clustering as the main functions, and those in rhizospheric soil showed metabolic clustering as the main function (Figure 6D).

## 4 Discussion

Over the past few years, the plant-microbiome association has received increasing attention for promoting beneficial interactions between plants and microorganisms to enhance specific outcomes for more sustainable agriculture (Basiru et al., 2021). Previous studies have shown that changes in the diversity or activity of plant symbiotic communities can significantly affect plant growth and environmental adaptation (Vandenkoornhuyse et al., 2015). Microbial communities usually vary according to plant species/cultivars, and the structure of microbial communities also depends on many environmental factors such as climatic conditions, water properties, and biological interactions (Fortin Faubert et al., 2022). Endophytic microorganisms vary in different tissues of plants (Wang et al., 2019). Beckers et al. (2017) believed that the endophytic bacterial communities in roots, stems, and leaves are highly variable as compared to the rhizospheric bacterial communities, and that each plant compartment represents a unique ecological niche for the bacterial community.

### 4.1 Changes in symbiotic- microbial diversity of *Schisandra chinensis*

In the present study, we examined the microbial communities in *S. chinensis* from different geographical locations, of different varieties, and in compartments with different ecological niches. The diversity and homogeneity of soil microbial community structure affect ecosystem stability; improve the ability of crops to respond to changes in the soil microbiological environment, particularly to pathogenic microorganisms; and enhance disease resistance of crops (Yang S. et al., 2022; Yang J. et al., 2022). The alpha-diversity analysis showed that the diversity and homogeneity of the microbial community were higher than those of the microbial community in the rhizospheric soil samples of *S. chinensis* and showed a highly significant relationship between different compartments. Following induction by root secretions and rhizospheric sediments, the soil microbiota first migrated to and concentrated around the rhizospheric area of the host. After host selection, some rhizosphere-inhabiting microorganisms penetrate the host roots, gradually colonize the internal tissues (horizontal spread), and further spread to the aboveground parts of the host (stems, flowers, and seeds; vertical spread) (Fresno and Munné-Bosch, 2021). We found that the diversity of microbial communities in *S. chinensis* samples decreased from underground to aboveground parts. This indicates that the physical barrier of the plant and the immune response induced by endogenous phytohormones regulate the microbial community (Zheng and Gong, 2019). In contrast, no significant difference was observed in the diversity of microbial communities of *S. chinensis* samples from different geographical locations, thus suggesting similar microbial communities in *S. chinensis* grown in different soil microenvironments (Qin et al., 2022). The homogeneity of the bacterial community of *S. chinensis* was a significant factor. The microbial communities in wild-type *S. chinensis* samples were more diverse and homogeneous than those in cultivated *S. chinensis* samples; moreover, the bacterial microbiota was more diverse in wild-type plants than in cultivated plants (Dutta et al., 2021). Beta-diversity analysis showed a high similarity of bacterial communities in the underground portion of *S. chinensis* samples, a high similarity of bacterial communities in stems and leaves in the aboveground portion, and a low similarity of bacterial communities in the fruit portion; this pattern may reflect the well-known selective gradient that exists from root to soil habitats (Fortin Faubert et al., 2022). In contrast, with regard to the fungal community, the five compartments (soil, roots, stems, leaves, and fruits) showed both similarity and distinctiveness; this showed that the fungal community was more influenced by the host plant than by the bacterial community (Liu et al., 2022).

### 4.2 Key taxa of symbiotic microorganisms of *Schisandra chinensis*

In the present study, the results of relative abundance between the compartments of *S. chinensis* samples showed that Proteobacteria was the dominant phylum (>72%) in the bacterial community of all *S. chinensis* samples and Ascomycota was the dominant phylum (>79%) in the fungal community of all *S. chinensis* samples. In the study of Qin Dan, the dominant bacteria also included *Cyanobacteria*, with a high abundance in roots, stems, leaves, and fruits (Qin et al., 2022). At the genus level, *Methylobacterium-Methylorubrum* and *Sphingomonas* were the dominant genera among the bacteria in all *S. chinensis* samples, and *Cladosporium* and *Uwebraunia* were the dominant genera among the fungi in all *S. chinensis* samples. The widespread presence of *Sphingomonas* and *Methylobacterium-Methylorubrum* is considered crucial in plants because of their multiple functions, including degradation of certain pollutants and enhancement of soil nutrient cycling and plant growth (Anguita-Maeso et al., 2019). According to previous studies, *Sphingomonas* plays a critical role in plant stress tolerance, plant growth promotion, and biodegradation of polycyclic aromatic hydrocarbons (Asaf et al., 2020). Leaves are more frequently exposed to harsh environmental conditions, including nutrient stress, desiccation, and UV radiation, which provide unique habitats for microorganisms (Hunter et al., 2010). *Methylobacterium-Methylorubrum* is usually isolated from the surfaces and interiors of leaves and can specifically colonize plants by utilizing methanol released by the plant (Galbally and Kirstine, 2002). *Methylobacterium-Methylorubrum* is a drought- and radiation-resistant species (Kim et al., 2019); hence, it can successfully and extensively colonize leaves (Lei et al., 2021). This finding is consistent with the high relative abundance of *Methylobacterium-Methylorubrum* in leaves in the present study. *Cladosporium* is a common fungal contaminant of herbal medicine (Yu et al., 2022), and it usually causes rot and plant diseases (Frasz and Miller, 2015). The frequent occurrence of decay and diseases in mature *S. chinensis* fruits might be related to their relatively high abundance of *Cladosporium*. Additionally, *Cladosporium* species may also pose a health risk, for example, spores of *Cladosporium* species can cause allergic diseases or sensitization (Sequino et al., 2022). *Uwebraunia*, an ectomycorrhizal fungus, is a member of the class Dothideomycetes; it can grow on other fungi (*Cryomyces*, *Coniosporium*, and *Friedmanniomyces*) and various substrates such as soil and rocks and can tolerate extreme climatic conditions (Haridas et al., 2020). This possibly explains the relatively high abundance of *Uwebraunia* in cultivated *S. chinensis*. The relative abundance of the dominant bacterial phyla was also inconsistent in the different organs of *S. chinensis*. Taken together, host selection (i.e., compartmentalized ecological niches and host species) is the predominant factor in shaping plant-microbiome assembly (Xiong et al., 2021). In this approach, host plants use secretions to recruit, filter, and enrich microbial taxa with specific functions in different ecological niches (Sasse et al., 2018).

We then conducted LEfSe analysis to identify microbial taxa significantly enriched in *S. chinensis* samples. The results showed that the microbial taxa of *S. chinensis* samples were more influenced by the compartments and less influenced by the geographical location and *S. chinensis* species. All five compartments of *S. chinensis* were significantly enriched with different microbial taxa. For example, *Actinobacteriota* was significantly enriched in rhizospheric soil-associated bacteria in *S. chinensis* samples. The Actinobacteriota includes many members of plant growth-promoting bacteria (Dawkins and Esiobu, 2018); these members can degrade plant residues in soil and convert them into an inorganic form that is more readily absorbed by plants (Wang X. et al., 2022; Wang Q. et al., 2022). *Basidiomycota*, a significantly enriched group of related fungi in leaves, can degrade low-quality substrates and recalcitrant lignin and lignocellulosic matrices (Yang S. et al., 2022; Yang J. et al., 2022). Based on the random forest analysis, we identified marker species at the genus level in different *S. chinensis* samples. We found more marker species in the stems and leaves of *S. chinensis*. In the rhizospheric soil samples of bacterial communities, *Nitrospira*, a well-known nitrite oxidizer present in the agricultural soil and rhizospheric soil of plants, is a significant marker species group (Cangioli et al., 2022). *Bradyrhizobium* present in roots is a beneficial microorganism for plants. *Bradyrhizobium* can overcome host plant defense and may survive as a chemo-organotrophic organism in rhizomes (Mayhood and Mirza, 2021). The marker populations of the fungal community showed differences mainly in the aboveground and belowground parts. *Penicillium* from *S. chinensis* samples was the marker population in the rhizospheric soil and root samples in the belowground portion; these fungal species possess various plant growth-promoting properties (Ismail et al., 2021). *Golubevia* was the marker population in stem and leaf samples from the aboveground parts. A recent study reported antagonistic interactions between *Golubevia heteromorpha* (cited as *Tilletiopsis pallescens*) and powdery mildew *Blumeria graminis* f. sp. *tritici* (Richter et al., 2019). Furthermore, although *Cladosporium* species are marker populations in fruit samples from the medicinal parts of *S. chinensis*, they frequently occur as secondary invaders of saprophytic or follicular lesions with other phytopathogenic fungi (Sandoval-Denis et al., 2015). These differences in marker species are closely associated with the different functions of the various compartments, thus suggesting that the compartments play a role in microbial selection (Xing et al., 2023). *S. chinensis* samples from different geographical locations, however, showed similarities in the core microbial communities of the same tissue. Therefore, we hypothesize that (1) geographic location does not significantly affect the endophytic microbial communities of *S. chinensis*, (2) the main differences are reflected in the ecological niches of *S. chinensis* itself, and (3) these microbial communities may play a key role in stabilizing the ecosystem function of *S. chinensis*.

### 4.3 Prediction of potential functions of *Schisandra chinensis* pombe symbiotic microorganisms

Plant secondary metabolites play a very important role in plant-pathogen interactions and plant defense (Fang et al., 2015). Qin Dan investigated the functions of the bacterial community in *Schisandra sphenanthera* and *Kadsura angustifolia*, which mainly included several critical aspects such as genetic information, human diseases, environmental information, cellular information, metabolism, and synthesis of organic substances (Qin et al., 2022). In the present study, we found that the main functions of the *S. chinensis* bacterial community (level 1) were biosynthesis, degradation/utilization/assimilation, detoxification effects, generation of precursor metabolites and energy, glycan pathway, macromolecule modification, and metabolic clustering. Higher abundance of the biosynthetic pathway of fatty acid (level 2) enrichment in *S. chinensis* symbiotic microbial communities, which is related to carbohydrate metabolism, amino acid metabolism and lipid metabolism, and is an important pathway for the production of volatile flavor substances (Song et al., 2022). In addition, there are also symbiotic microorganisms enriched to the tricarboxylic acid cycle pathway. The metabolites of the tricarboxylic acid cycle are simple organic acids, which play a role in many physiological processes (Fan et al., 2021).

## 5 Conclusion

In conclusion, this study investigated plant microbial communities in several phytoecological niches and geographic locations of *S. chinensis*. The results suggest that plant organs have an important regulatory role in the composition and structure of symbiotic microorganisms. Moreover, although the microbial communities of *S. chinensis* from different geographic locations were similar in the same tissue, their abundance changed according to the geographic locations. These findings provide new insights into the distribution and resources of plant symbiotic microorganismsin different ecological niches and offer new opportunities to gain knowledge related to plant microbial communities and potential functions.

## Data availability statement

The datasets presented in this study can be found in online repositories. The names of the repository/repositories and accession number(s) can be found in the article/Supplementary material.

## Author contributions

WH: Writing – review & editing, Writing – original draft. YX: Software, Writing – review & editing. HX: Resources, Writing – review & editing. YaH: Resources, Writing – review & editing. YuH: Formal analysis, Writing – review & editing. WM: Writing – review & editing. YY: Conceptualization, Investigation, Writing – review & editing. TK: Software, Writing – review & editing. DD: Writing – review & editing. HZ: Supervision, Writing – review & editing. LX: Conceptualization, Investigation, Writing – review & editing.

## Footnotes

^1^ https://www.genescloud.cn

^2^ https://metacyc.org/
