# Supplementary material for: Exploring the diversity and potential functional characteristics of microbiota associated with different compartments of Schisandra chinensis
Source: Front Microbiol. 2024 Jun 13;15:1419943. doi: 10.3389/fmicb.2024.1419943 (PMC11208631; doi:10.3389/fmicb.2024.1419943)
Supplement: Supplementary file 1 [file Data_Sheet_1.docx]

**Exploring the diversity and potential functional characteristics of microbiota associated with different compartment of *Schisandra chinensis***

Wenjuan Hou^1†^, Yanping Xing^1†^, Hefei Xue^1^, Yanchang Huang^1^, Yutong Huang^1^, Wenxiao Men^1^, Yanyun Yang^1,2^*, Tingguo Kang^1^, Deqiang Dou^1^, Han Zheng^2^, Liang Xu^1,2^*

^1^School of Pharmacy, Liaoning University of Traditional Chinese Medicine, Dalian, China

^2^State Key Laboratory of Dao-di Herbs，Beijng

***** Corresponding authors: Yanyun Yang [999yyy@163.com](mailto:999yyy@163.com); Liang Xu [861364054@qq.com](mailto:861364054@qq.com); Telephone and fax numbers: 0086411-85890128 (Yanyun Yang and Liang Xu)

Equal contributions: Wenjuan Hou^1†^ and Yanping Xing^1†^ These authors contributed equally to this work and share first authorship

**Table S1** Information Sheet for Sample Collection of *Schisandra chinensis*

| No. | Sites | Longitude and Latitude | Collection time | Growing year |
| --- | --- | --- | --- | --- |
| 1 | Qianshan, Liaoning, China | E123°9′1.16′′ N41°0′55.1′′ | 2023-9 | Null |
| 2 | Zhuanghe City, Liaoning Province, China | E122°57′20.4′′ N39°46′2 5.1′′ | 2023-9 | 4 |
| 3 | Fushun City, Liaoning Province | E124°49′56′′ N41°42′48′′ | 2023-9 | 4 |
| 4 | Benxi City, Liaoning | E125°2′13′′ N41°23′47′′ | 2023-9 | 5 |
| 5 | Fengcheng City, Liaoning Province | E124°7′42′′ N40°30′60′′ | 2023-9 | 4 |
| 6 | Kandian County, Liaoning Province | E124°46′41′′ N40°43′46′′ | 2023-9 | 5 |

**Table S2** Sequencing data for rhizospheric soil, roots, stems, leaves and fruits

| Sample ID | Tissue | Bacterial sequences | Fungal sequences | Bacterial ASVs | Fungal ASVs |
| --- | --- | --- | --- | --- | --- |
| QT1 | rhizospheric soil | 121085 | 89623 | 31880 | 76266 |
| QT2 | rhizospheric soil | 118625 | 88178 | 34473 | 78216 |
| QT3 | rhizospheric soil | 109696 | 88053 | 29242 | 69233 |
| QTG1 | Root | 117704 | 81440 | 43879 | 71667 |
| QTG2 | Root | 117564 | 83873 | 38552 | 70034 |
| QTG3 | Root | 112502 | 71073 | 33100 | 65581 |
| QJ1 | Stem | 108091 | 80177 | 41649 | 58308 |
| QJ2 | Stem | 122817 | 84573 | 46544 | 63492 |
| QJ3 | Stem | 115700 | 88139 | 53909 | 69493 |
| QY1 | Leaf | 114191 | 95502 | 46356 | 73708 |
| QY2 | Leaf | 112201 | 91938 | 55075 | 79533 |
| QY3 | Leaf | 117557 | 96896 | 48151 | 83618 |
| QG1 | Fruit | 109176 | 107426 | 52733 | 81608 |
| QG2 | Fruit | 102587 | 116313 | 89972 | 112433 |
| QG3 | Fruit | 111006 | 92540 | 69378 | 82313 |
| ZT1 | rhizospheric soil | 126574 | 80105 | 40570 | 67485 |
| ZT2 | rhizospheric soil | 123663 | 80034 | 37479 | 62078 |
| ZT3 | rhizospheric soil | 116428 | 83451 | 29970 | 69715 |
| ZTG1 | Root | 113845 | 80876 | 31868 | 61282 |
| ZTG2 | Root | 113026 | 79377 | 56948 | 55923 |
| ZTG3 | Root | 115971 | 78411 | 44170 | 65541 |
| ZJ1 | Stem | 106670 | 70988 | 43082 | 56319 |
| ZJ2 | Stem | 104112 | 77897 | 39420 | 56380 |
| ZJ3 | Stem | 114471 | 92717 | 43214 | 78621 |
| ZY1 | Leaf | 103242 | 101839 | 49625 | 89314 |
| ZY2 | Leaf | 93054 | 86719 | 41289 | 74250 |
| ZY3 | Leaf | 94333 | 96535 | 52216 | 80912 |
| ZG1 | Leaf | 81414 | 77163 | 72824 | 73472 |
| ZG2 | Leaf | 82639 | 89736 | 52610 | 85457 |
| ZG3 | Leaf | 84634 | 74825 | 70020 | 70836 |
| XT1 | rhizospheric soil | 112250 | 81634 | 37481 | 70755 |
| XT2 | rhizospheric soil | 105186 | 76488 | 37877 | 61805 |
| XT3 | rhizospheric soil | 105247 | 85447 | 36274 | 71504 |
| XTG1 | Root | 98230 | 85908 | 37726 | 75000 |
| XTG2 | Root | 106990 | 91340 | 44979 | 68658 |
| XTG3 | Root | 101866 | 104948 | 41961 | 80637 |
| XJ1 | Stem | 101650 | 101971 | 38303 | 81914 |
| XJ2 | Stem | 101584 | 98282 | 39983 | 82600 |
| XJ3 | Stem | 91512 | 83922 | 44613 | 67301 |
| XY1 | Leaf | 99822 | 79359 | 42820 | 64078 |
| XY2 | Leaf | 98203 | 104528 | 39492 | 80402 |
| XY3 | Leaf | 94969 | 80342 | 52438 | 67412 |
| XG1 | Fruit | 80975 | 109128 | 72092 | 102143 |
| XG2 | Fruit | 85700 | 110739 | 76324 | 103670 |
| XG3 | Fruit | 97547 | 106405 | 45287 | 99445 |
| FT1 | rhizospheric soil | 102517 | 88334 | 32305 | 69781 |
| FT2 | rhizospheric soil | 103774 | 96195 | 32123 | 80946 |
| FT3 | rhizospheric soil | 96022 | 109930 | 37455 | 93022 |
| FTG1 | Root | 128311 | 92178 | 50339 | 76710 |
| FTG2 | Root | 104956 | 79432 | 49853 | 66178 |
| FTG3 | Root | 106512 | 87710 | 43472 | 74511 |
| FJ1 | Stem | 109522 | 84598 | 48507 | 70248 |
| FJ2 | Stem | 110290 | 85041 | 53030 | 74206 |
| FJ3 | Stem | 111511 | 97284 | 60730 | 87760 |
| FY1 | Leaf | 118338 | 102903 | 70582 | 87432 |
| FY2 | Leaf | 108775 | 91393 | 55392 | 75226 |
| FY3 | Leaf | 109298 | 104195 | 64690 | 96197 |
| FG1 | Fruit | 117064 | 87965 | 80878 | 47272 |
| FG2 | Fruit | 121077 | 86580 | 66202 | 45510 |
| FG3 | Fruit | 90581 | 88356 | 38711 | 47718 |
| KT1 | rhizospheric soil | 113018 | 98359 | 30255 | 76437 |
| KT2 | rhizospheric soil | 110563 | 83133 | 31243 | 65432 |
| KT3 | rhizospheric soil | 111798 | 88236 | 32514 | 70909 |
| KTG1 | Root | 108118 | 88211 | 80256 | 68519 |
| KTG2 | Root | 104718 | 85102 | 81278 | 47606 |
| KTG3 | Root | 124062 | 84306 | 48095 | 66542 |
| KJ1 | Stem | 116546 | 88656 | 57152 | 79250 |
| KJ2 | Stem | 112208 | 88970 | 43989 | 75156 |
| KJ3 | Stem | 122520 | 87956 | 76047 | 78375 |
| KY1 | Leaf | 112476 | 84219 | 48355 | 70889 |
| KY2 | Leaf | 116996 | 95079 | 61072 | 81449 |
| KY3 | Leaf | 120709 | 89327 | 82063 | 77157 |
| KG1 | Fruit | 123372 | 112840 | 97335 | 106278 |
| KG2 | Fruit | 112753 | 110820 | 98802 | 104125 |
| KG3 | Fruit | 122659 | 105336 | 108678 | 98933 |
| HT1 | rhizospheric soil | 112202 | 85185 | 35827 | 65096 |
| HT2 | rhizospheric soil | 114284 | 80461 | 40100 | 65288 |
| HT3 | rhizospheric soil | 112211 | 75462 | 40556 | 62176 |
| HTG1 | Root | 132574 | 80093 | 56173 | 67246 |
| HTG2 | Root | 125667 | 73873 | 45389 | 60943 |
| HTG3 | Root | 134393 | 81461 | 58767 | 64649 |
| HJ1 | Stem | 108884 | 89220 | 70171 | 79815 |
| HJ2 | Stem | 119485 | 74413 | 67839 | 64478 |
| HJ3 | Stem | 115324 | 74601 | 57015 | 61659 |
| HY1 | Leaf | 116598 | 114474 | 56005 | 96200 |
| HY2 | Leaf | 136832 | 115135 | 71513 | 81179 |
| HY3 | Leaf | 117135 | 92077 | 57980 | 73599 |
| HG1 | Fruit | 94297 | 80318 | 86507 | 76967 |
| HG2 | Fruit | 106637 | 99085 | 86356 | 94582 |
| HG3 | Fruit | 78420 | 71914 | 72546 | 68751 |

Q: samples from Qianshan; Z: samples from Zhuanghe; X: samples from Xinbin; F: samples from Fengcheng; K: samples from Kuandian; H: samples from Huanren; T: samples ecotopes are rhizospheric soils; TG: samples ecotopes are roots; J: samples ecotopes are stems, L: samples ecotopes are leaves and G: samples ecotopes are fruits.


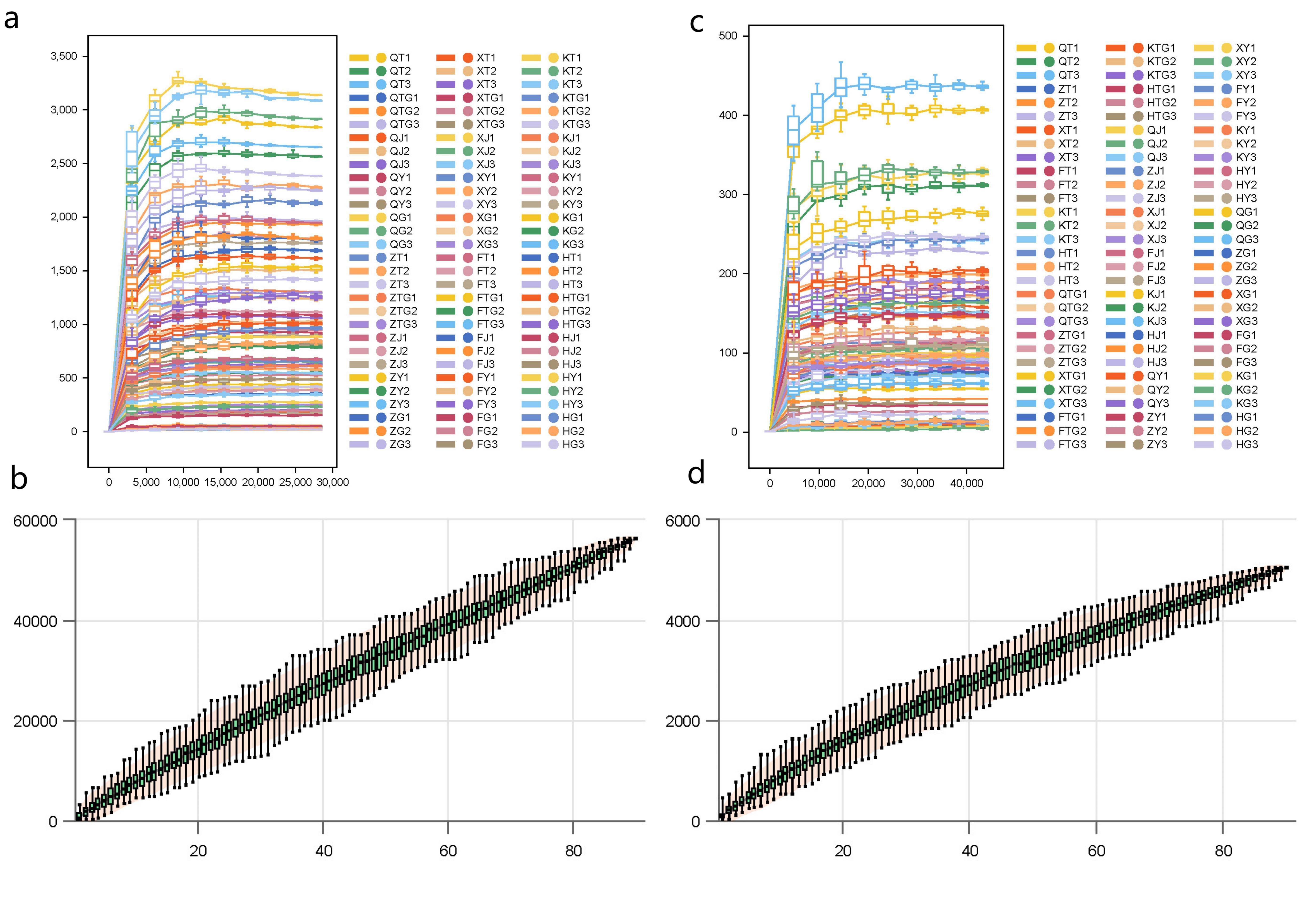


**Figure S1** a and b represent the dilution curves and species accumulation curves for the bacterial community of the *Schisandra chinensis* samples. c and d represent the dilution curves and species accumulation curves for the fungal community of the *Schisandra chinensis* samples.


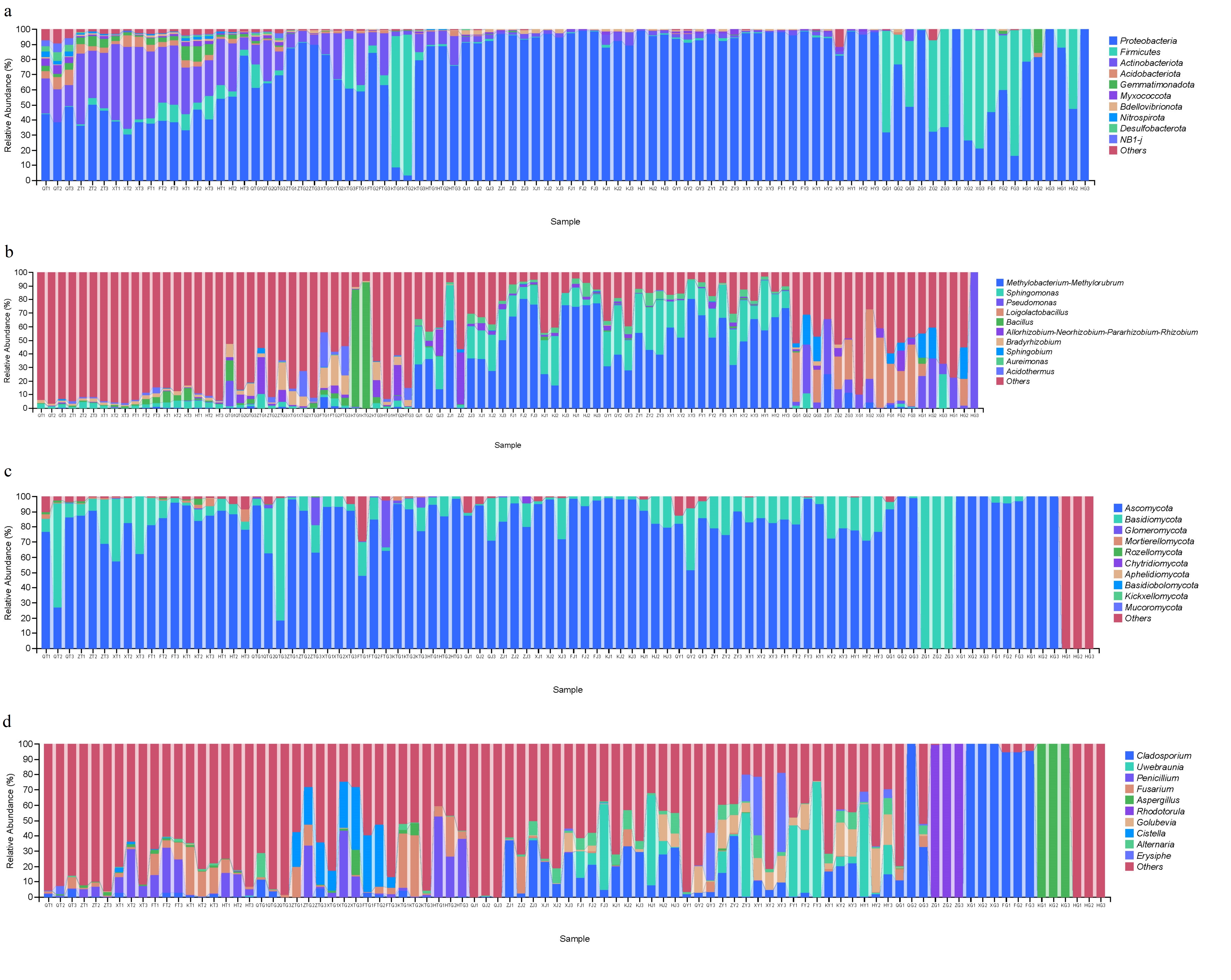


**Figure S2** Relative abundance in all *Schisandra chinensis* samples. (a-b) denote bacterial communities, (c-d) denote fungal communities, a and c denote phylum level, and b and d denote genus level.


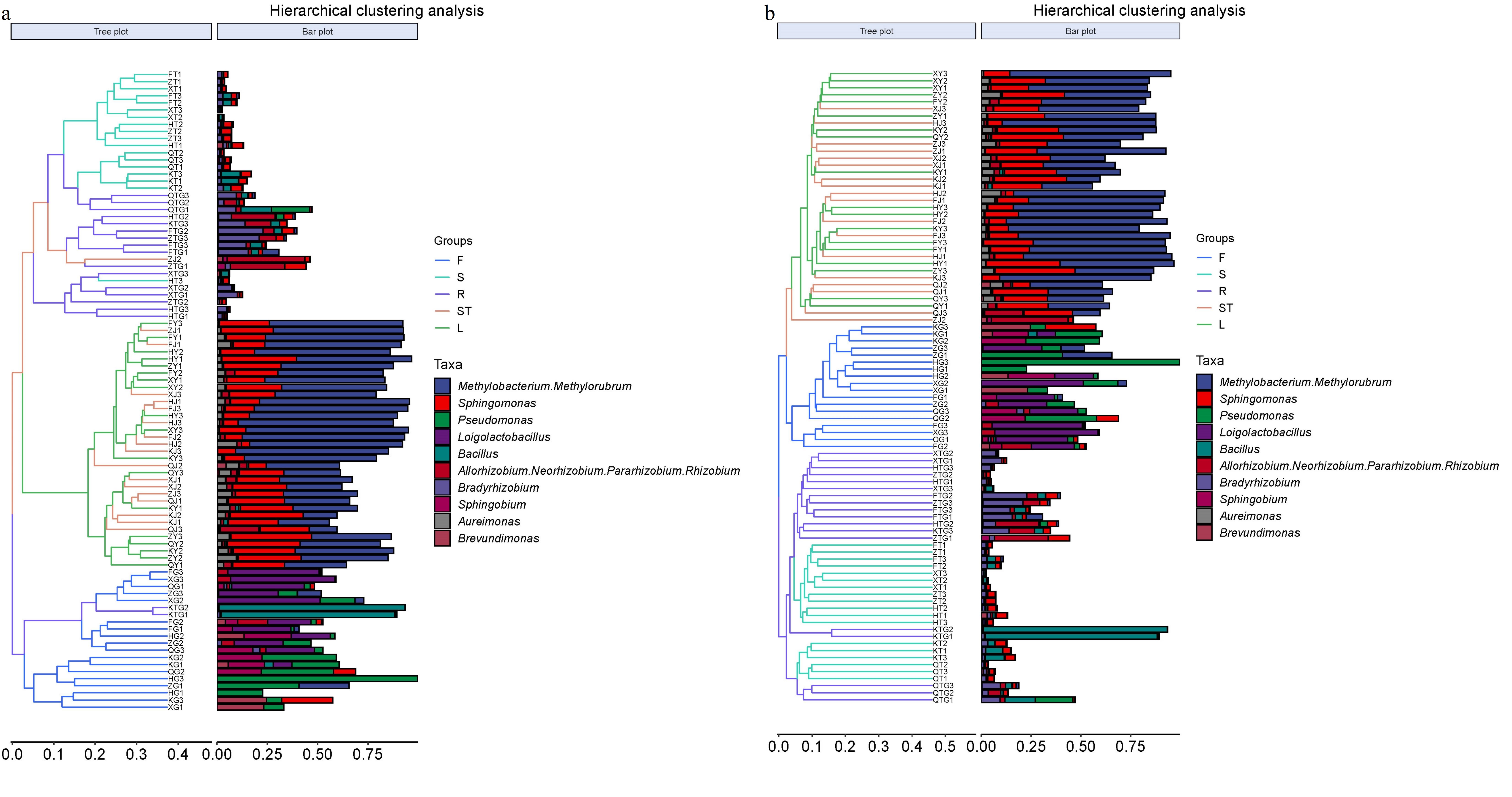


**Figure S3** Hierarchical clustering analysis was done with Weighted Unifrac distance matrix and UnWeighted Unifrac distance matrix. (a) represents Weighted Unifrac distance matrix，(b) represents UnWeighted Unifrac distance matrix.





**Figure S4** Hierarchical clustering of top 10 genera bacterial (a-b) and the fungal communities (c-d). a and c denote hierarchical clustering across geographic locations. b and d denote hierarchical clustering of different organs.
